# Implementation and maintenance of a pain management quality assurance program at intensive care units: 360 degree feedback of physicians, nurses and patients

**DOI:** 10.1371/journal.pone.0208527

**Published:** 2018-12-19

**Authors:** Christian Smolle, Gerald Sendlhofer, Andreas Sandner-Kiesling, Michael K. Herbert, Lydia Jantscher, Bernd Pichler, Lars-Peter Kamolz, Gernot Brunner

**Affiliations:** 1 Research Unit Safety in Health, c/o Division of Plastic, Aesthetic and Reconstructive Surgery, Department of Surgery, Medical University of Graz, Graz, Austria; 2 Division of Plastic, Aesthetic and Reconstructive Surgery, Department of Surgery, Medical University of Graz, Graz, Austria; 3 Executive Department for Quality and Risk Management, University Hospital Graz, Graz, Austria; 4 Department of Anaesthesiology and Intensive Care Medicine, Medical University of Graz, Graz, Austria; University of Antwerp, BELGIUM

## Abstract

**Background:**

Pain management quality assurance programs (PMQP) have been successfully implemented in numerous hospitals across Europe. We aimed to evaluate the medium-term sustainability of a PMQP implemented at intensive care units (ICUs).

**Methods:**

Two surveys, the first in 2012, immediately after introduction of the PMQP, and the second in 2015, were carried out amongst patients, physicians and nurses. Demographic parameters of all participants were assessed. Patients were asked after their pain levels during ICU stay. Staff members answered a questionnaire regarding familiarity with standards and processes of PMQP and self-perception of their knowledge as well as contentment with interdisciplinary communication.

**Results:**

In total (2012/2015), 267 (125/142) patients, 113 (65/48) physicians and 510 (264/246) members of the nursing staff participated. Minimum and maximum pain levels of patients did not differ between both surveys. Patients’ tolerance of pain 24 hours before the survey was better (p = 0.023), and vomiting occurred less often (p = 0.037) in 2015. Physicians’ and nurses’ contentment with the own knowledge about pharmacological pain treatment had increased from 2012 to 2015 (p = 0.002 and 0.004). Satisfaction with communication between nurses and physicians was better in 2015 (p<0.001 and p = 0.002). Familiarity with PMQP standards and processes remained stable in both collectives.

**Conclusion:**

The implementation of our PMQP achieved a high standard of care, guarantying a high patient and staff member satisfaction. Continuous education, ongoing training, regular updates and implementation of feedback-loops ensure continuity, in some parameters even an increase in knowledge and competencies. This is mirrored in high patient and staff member satisfaction.

## Introduction

Quality management concepts for acute pain therapy have successfully been implemented in numerous hospitals in Europe recently [1–6]. Evidence-based clinical practice guidelines for pain management improve pain management towards higher efficacy, which is on the one hand reflected in improved analgesia, but also in significantly increased patient satisfaction [2, 5, 7–9]. Despite thorough integration of all professions, achievements made by implementation of new standards and processes may however be difficult to sustain and long-lasting effects cannot always be ensured. In part this can be due to poor adherence to clinical guidelines, where the beneficial potential of novel concepts may even be compromised by a negative attitude of the personnel [1, 10–12].

Following a process of evaluation, a pain management quality assurance program (PMQP) was introduced in all 16 departments of the hospital of the Medical University of Graz between 2009 and 2012 according to the certification criteria of “Certkom”, Germany. Before that, pain management, including assessment and subsequent therapeutic measures, was carried out unstructured and more or less according to the individual experience and preferences of the attending physicians and nurses, resulting in vast differences in the treatment of pain conditions. Our PMQP included the implementation of interdisciplinary working groups for pain management, of evidence-based clinical practice guidelines for pain treatment, of comprehensive advanced educational curricula and trainings and of thorough assessment, documentation and treatment of patients’ pains. The established regimens for pain treatment were adapted considering the pathophysiology of the underlying disease and accompanying illnesses, plus the type and extent of any given medical intervention. Our pharmacological pain regimens included both baseline and on-demand-medication. Treatment of side effects like discomfort, constipation, dyspnoea, nausea or vomiting also followed a particular regimen adapted to the individual patient’s needs [13].

In critically ill patients adequate management of pain is crucial to ensure best possible clinical outcomes [14]. The aim of this study was to evaluate the efficacy and sustainability of the implementation of our PMQP at intensive care units (ICUs) through assessment of patient reported outcome during two periods. Furthermore, the attitude of the attending physicians and nurses towards the PMQP was evaluated. The intention was to provide an overview over the attitudes of all people involved into the PMQP thus receiving a “360 degree feedback”.

## Material and methods

The study has been approved by the institutional review board of the Medical University of Graz (vote#: 28–235 ex 15/16). As approved by the review board, written informed consent was obtained from all participants.

### Pain management quality assurance program (PMQP)

The PMQP strictly followed the certification criteria from “Certkom” (http://www.certkom.com) and included following dimensions of care quality:

1. Structure–includes all of the factors that affect the context in which care is delivered
2. Process–includes the sum of all actions that make up healthcare
3. Outcome–contains all the effects of healthcare on patients

To address all these aspects within the university hospital four main initiatives, namely

1. development of therapeutic standards and templates for pain-management documentation
  ○ development of standard operating procedures (SOPs)
  ○ development of advanced educational curricula, and training as well as
  ○ comprehensive patient information

were implemented and certified by”Certkom”.

The development of therapeutic standards and corresponding SOPs were performed by a multidisciplinary work group consisting of physicians, nurses and therapists and were led by an expert on pain management from the anesthesiological department and moderated by Executive Department for Quality and Risk Management. Therapeutic standards followed the WHO model analgesic ladder [15], and were printed on small cards which were handed out to the attending staff.

Accompanying SOPs were developed and trained to all healthcare professionals and included i) admission and discharge of patients, ii) information, guidance and training of patients’ pain, iii) administration of opioids, iv) pain management measurement, v) documentation of pain, vi) non-pharmacological options of pain management as well as vii) standard treatment of nausea and vomiting.

Alongside, an educational curriculum for all healthcare professionals group was developed in order to guarantee concomitant education of SOPs and from time to time refreshing of pain therapy knowledge. These curricula are offered each year and physicians and nurses are obliged once in two years to take part. For new employees, additional enrolment trainings are offered and documented in a personal training log. The external certifier not only proofs the three dimension of quality, they also proof, if training and education was performed properly. Furthermore, in order to also address the patient, information leaflets in several languages were developed and distributed on each ward.

Internal audits and patient and employee surveys were used to assure adherence to the implemented PMQP. The subjective wellbeing of patients treated at nine ICUs of the University Hospital of the Medical University of Graz was assessed prospectively. Simultaneously, we collected data on the attending physicians’ and nurses’ attitude towards the PMQP. Finally, in 2012 the PMQP was certified by Certkom. The evaluations ran during two consecutive periods, the first from January 12^th^ until January 30^th^, 2012 (immediately after implementation of the PMQP), and the second from October 8^th^ until October 20^th^, 2015. In-between this time periods, it was the effort to keep implemented PMQP on a high level in order to guarantee compliance and maintenance. Therefore, internal audits were put in place to regularly support physicians and nurses with a feedback on the pain management process. Internal audits focussed on adherence of SOPs, pain management documentation, pain therapy and if regular trainings were performed by screening the training log file.

### Patients’ satisfaction

The participating patients came from surgical departments, including general surgery, thoracic surgery, vascular surgery, orthopaedics and trauma and neurosurgery, and from the non-surgical departments internal medicine and neurology. All patients staying at the ICU during the respective study periods were asked to participate in the survey. Written informed consent, demographic and treatment parameters were ascertained from each patient. They answered a questionnaire concerning both pain and analgesic treatment effects and side-effects they had experienced during their stay as shown in Table 1. Participants unable to handle the form by themselves due to their medical condition were interviewed by an attending staff member who filled in the questionnaire for them. Patients who were unable to actively take part in the survey, i.e. fill out the questionnaire, due to illness (mechanical ventilation, neurological condition, etc.) were not excluded from the survey. In such cases nurses filled out the survey in accordance to the patients’ answers. Only patients who were more than 2 days at one of the ICUs were included into the survey. In 2012, in total 225/340 eligible patients took part (overall 66.2%, surgical ICUs: n = 164; non-surgical ICU: n = 48; Stroke Unit: n = 13). In 2015, in total 147/229 eligible patients took part (overall 64.2%, surgical ICUs: n = 109; non-surgical ICU: n = 21; Stroke Unit: n = 17).

**Table 1:** Questionnaire for patients.

Questions
1. What was the most severe pain you have experienced since admission? (numeric rating scale [NRS] 0–10; 0 = no pain, 10 = worst imaginable pain)
2. What was the minimal pain you have experienced since admission? (NRS 0–10)
3. How bearable was the pain during the last 24 hours? (scale 0–10; 0 = well bearable, 10 = not bearable)
4. How do you judge the efficacy of your pain medication? (scale 0–10; 0 = ineffective, 10 = very effective)
5. Were you encouraged to let the staff know when you had pain? (yes/no/do not know)
6. Regardless of whether you received any, would you have wanted more pain medication? (yes/no/do not know)
7. Did you suffer from any of the following symptoms during your stay? (possible answers: yes/no/do not know) • Nausea • Vomiting • Itching • Breathlessness • Sleeping problems • Anxiety • Helplessness

Age, gender, presence of a malignancy, surgical or conservative management of the underlying disease and need for artificial ventilation or catecholamine requirement were documented of each patient. Regarding pain management, the administration of oral analgesics, parenteral analgesics or transdermal analgesics, and the use of regional anaesthesia was assessed. Orally and parenterally administered analgesics were further categorized according to the WHO analgesic ladder (WHO 1: non-opioid analgesics, WHO 2: weak opioids, WHO 3: strong opioids) [15].

### Physicians’ knowledge

Age, gender and working place (surgical or conservative ICU) were assessed. Each physician working in the respective ICU was asked to participate in the survey and to answer a questionnaire focusing on both the familiarity with the PMQP items and on the subjective appraisal of PMQP standards and processes as shown in Table 2. In 2012 and 2015, 85 and 64 physicians participated in the survey (response rates 68% and 61%), respectively.

**Table 2:** Questionnaire for physicians and nurses.

Physicians-Questionnaire	Nursing-Questionnaire
1. At your ward, is there a multidisciplinary work group for pain management that includes members of the nursing staff, physicians and physiotherapists? (yes/no/do not know)	1. At your ward, is there a multidisciplinary work group for pain management that includes members of the nursing staff, physicians and therapists? (yes/no/do not know)
2. At your ward, is there a written consent about the responsibilities in pain management? (yes/no/do not know)	2. At your ward, is there a written consent about the responsibilities in pain management? (yes/no/do not know)
3. Are written standards available that you can access when you face a problematic pain situation in a patient? (yes/no/do not know)	3. At your ward, are there medical standards that regulate medicinal treatment of patients? (yes/no/do not know)
4. Is there a written standard for the treatment of nausea and vomiting? (yes/no/do not know)	4. Is there a written standard for treatment of nausea and vomiting? (yes/no/do not know)
5. Is there a written standard for the treatment of constipation? (yes/no/do not know)	5. Is there a written standard for treatment of constipation? (yes/no/do not know)
6. Is there a written standard for sedation? (yes/no/do not know)	6. Is there a written standard for sedation? (yes/no/do not know)
7. Is there a written standard for non-pharmacological options of pain management? (yes/no/do not know)	7. Is there a written standard for non-pharmacological options of pain management? (yes/no/do not know)
8. (-)	8. Is there a written standard for preventive pharmacological pain therapy before nursing interventions? (yes/no/do not know)
9. (-)	9. When you call a physician for a drug prescription, how long do you have to wait for it? a. Within 15 minutes b. After more than 15 minutes c. After more than 30 minutes d. After more than 60 minutes
10. (-)	10. Do you attend pain management training at least once a year? (yes/no)
11. (-)	11. Do you use transfer protocols that take the patient’s individual pain management into account? (yes/no/do not know)
12. How satisfied are you with… (Likert scale 1–5; 1 = very content, 5 = not at all content)	12. How satisfied are you with… (Likert scale 1–5, 1 = very content, 5 = not at all content)
a. …your own knowledge about pharmacological pain management?	a. …your own knowledge about pharmacological pain management?
b. …your own knowledge about non-pharmacological options?	b. …your own knowledge about non-pharmacological options?
c. …the nursing staff’s knowledge about pain management?	c. …the physicians’ knowledge about pain management?
d. …the therapists’ knowledge about pain management?	d. …the therapists’ knowledge about pain management?
e. …communication between nurses and physicians?	e. …communication between nurses and physicians?
f. …communication between physicians?	f. …communication within the nursing staff?
g. …communication between physiotherapists and nurses?	g. …communication between physiotherapists and nurses?
h. …communication between physiotherapists and physicians?	h. …communication between physiotherapists and physicians?
i. …the pain management training offered at your department?	i. …the pain management training offered at your department?

### Nurses’ knowledge

Age, gender and years of working experience in intensive care were assessed for registered nurses. Each nurse working in the respective ICU was asked to participate in the survey and to answer the questionnaire as outlined for physicians. In 2012 and 2015, 357 and 227 registered nurses participated in the survey (response rates 69% and 56%), respectively. Further items of the evaluation included the specification of patients treated (surgical, conservative, mixed) and if a special intensive care training had been completed. Each nurse was asked to answer a questionnaire focusing on the familiarity with the PMQP items, on indicators of degree of implementation and on the subjective appraisal of PMQP as shown in Table 2.

### Statistical analysis

Statistical analysis was done with SPSS 23.0 for Windows (IBM Inc., Armonk, NY, USA). A p-value below 0.05 was considered statistically significant.

#### Univariate inductive analysis

The results of the two survey periods were compared. For binary parameters, Fisher’s Exact Test was used. Chi^2^-test was used for categorical data with more than two characteristics. T-test was applied for the comparison of continuous parameters, and Spearman’s correlation analysis was used for ordinal variables. Whenever present, the option “do not know” as well as missing values were excluded from descriptive analysis.

#### Linear regression models

Linear regression models were used to determine whether changes of the survey results were solely based on the survey period, or were affected by changes of basic demographic or treatment parameters. Questionnaire parameters were related to basic demographic and treatment parameters that showed significant differences between 2012 and 2015 as well as to the survey period itself (2012 or 2015).

## Results

### Patients

The 2012 and 2015 periods showed no differences regarding age or gender distribution. Of 225 patients in 2012, 162 answered the survey by themselves, 63 were answered by nurses. Of 147 patients in 2015, 111 answered the survey by themselves, 36 were answered by nurses. In 2015, less surgical patients attended the survey (2012: 80.0% vs. 2015: 67.6%, p = 0.026), whereas more patients required artificial ventilation during their ICU stay as shown in Table 3 (2012: 6.4% vs. 2015: 14.1%, p = 0.046). The 2015 cohort received non-opioids orally more often (2012: 11.2% vs. 2015: 24.6%, p = 0.001), otherwise no differences between the two periods regarding pharmacological pain treatment could be found as shown in Table 4.

**Table 3:** Sample characteristics of patients. No significant differences were observed concerning age or gender between the two survey periods, however, the share of surgical patients was smaller and the number of patients requiring artificial ventilation was greater in 2015.

Parameter (patients)	Patients, n (%)	Characteristics 2012	Characteristics 2015	Test, p-value
*Total (n)*	267	125	142	-
*Gender*	Male: 166 (62.2%)	Male: 79 (63.2%)	Male: 87 (61.3%)	
	Female: 101 (37.8%)	Female: 46 (36.8%)	Female: 55 (38.7%)	Fisher’s test, p = 0.801
*Mean age (years)*	64.4 (SD ±14.1)	64.4 (SD ±14.2)	64.4 (SD ±13.9)	T-test, p = 0.976
*Surgical (S) or conservative (C) management of disease*	S: 196 (73.4%)	S: 100 (80.0%)	S: 96 (67.6%)	
	C: 71 (26.6%)	C: 25 (20.0%)	C: 46 (32.4%)	Fisher’s test, p = 0.026
*Malignancy*	59 (22.1%)	21 (16.8%)	38 (26.8%)	Fisher’s test, p = 0.056
*Artificial ventilation during ICU stay*	28 (10.5%)	8 (6.4%)	20 (14.1%)	Fisher’s test, p = 0.046
*Administration of catecholamines during ICU stay*	25 (8.4%)	13 (10.4%)	12 (8.5%)	Fisher’s test, p = 0.675

**Table 4:** Pain medication administered during the two respective study periods. Parenteral WHO 1 pain medication was administered more frequently in the 2015 collective (Fisher’s test, p = 0.007), otherwise there were no significant differences.

Pain medication	Total (n = 267)	2012 (n = 125)	2015 (n = 142)	Test, p-value
Oral analgesics	77 (28.8%)	30 (24.0%)	47 (33.1%)	Fisher’s test, p = 0.107
WHO 1	49 (18.4%)	14 (11.2%)	35 (24.6%)	Fisher’s test, p = 0.007
WHO 2	8 (3.0%)	4 (3.2%)	4 (2.8%)	Fisher’s test, p = 1.000
WHO 3	20 (7.5%)	12 (9.6%)	8 (5.6%)	Fisher’s test, p = 0.250
Parenteral analgesics	196 (73.4%)	95 (76.0%)	101 (71.1%)	Fisher’s test, p = 0.406
WHO1	27 (10.1%)	10 (8.0%)	17 (12.0%)	Fisher’s test, p = 0.315
WHO2	12 (4.5%)	5 (4.0%)	7 (4.9%)	Fisher’s test, p = 0.775
WHO3	159 (59.6%)	80 (64.0%)	79 (55.6%)	Fisher’s test, p = 0.172
Transdermal analgesics (only WHO3)	4 (1.5%)	1 (0.8%)	3 (2.1%)	Fisher’s test, p = 0.625
Regional anaesthesia	3 (1.1%)	0 (0.0%)	3 (2.1%)	Fisher’s test, p = 0.250

Patients reported similar median most severe and minimal pain in both survey periods. Patients from 2015, however, stated that pain levels within 24 hours before the survey were more bearable (p = 0.023). Out of the assessed symptoms, vomiting occurred less often in the 2015 collective as shown in Table 5 (p = 0.037). Satisfaction with analgesic treatment was similar in both collectives, with a similar share reporting less than medium (<5 of 10 points) satisfaction with pain management (2012: 8.8% vs. 2015: 6.3%, p = 0.491).

**Table 5:** Survey results of patients. SD = Standard deviation, NRS = Numeric rating scale, results of questions 1–4 given in median values and range in brackets.

Item	Overall result (n = 267)	Result 2012 (n = 125)	Result 2015 (n = 142)	Test, P-value
*1*. *Most severe pain since admission*? *(NRS; 0 = no pain*, *10 = worst imaginable pain)*	5 [0–10], Missing: 1 (0.4%)	5 [0–10], Missing: 0	6 [0–10], Missing: 1 (0.7%)	Spearman-correlation, p = 0.466
*2*. *Minimum pain since admission*? *(NRS; 0 = no pain*, *10 = worst imaginable pain)*	0 [0–6], Missing: 1 (0.4%)	0 [0–6], Missing: 1 (0.8%)	0 [0–6], Missing: 1 (0.7%)	Spearman-correlation, p = 0.787
*3 How bearable was the pain during the last 24 hours*? *(0 = well bearable*, *10 = not bearable)*	2 [0–10], Missing: 2 (0.7%)	2 [0–10], Missing: 2 (1.6%)	2 [0–9], Missing: 0	Spearman-correlation, p = 0.023
*4*. *How do you judge the efficacy of your pain medication*? *(0 = ineffective*, *10 = very effective)*	10 [0–10], Missing: 4 (1.5%)	10 [1–10], Missing: 4 (3.2%)	10 [0–10], Missing: 0	Spearman-correlation, p = 0.322
*5*. *Were you encouraged to let the staff know when you had pain*? *(answer = yes)*	253 (94.8%)	117 (93.6%)	136 (95.8%)	Fisher’s test, p = 0.102
*6*. *Regardless of whether you received any*, *would you have wanted more pain medication*? *(answer = yes)*	28 (10.5%)	15 (12.0%)	13 (9.2%)	Fisher’s test, p = 0.554
7. Did you suffer from any of the following symptoms during your stay?				
*a*. *Nausea*	58 (21.7%)	30 (24.0%)	28 (19.7%)	Fisher’s test, p = 0.458
*b*. *Vomiting*	32 (12.0%)	21 (16.8%)	11 (7.7%)	Fisher’s test, p = 0.037
*c*. *Itching*	25 (9.4%)	12 (9.6%)	13 (9.2%)	Fisher’s test, p = 1.000
*d*. *Breathlessness*	65 (24.3%)	26 (20.8%)	39 (27.5%)	Fisher’s test, p = 0.254
*e*. *Sleeping problems*	120 (44.9%)	61 (48.8%)	59 (41.5%)	Fisher’s test, p = 0.174
*f*. *Anxiety*	58 (21.7%)	25 (20.0%)	33 (23.2%)	Fisher’s test, p = 0.551
*g*. *Helplessness*	28 (10.5%)	12 (9.6%)	16 (11.3%)	Fisher’s test, p = 0.690

The parameters “artificial ventilation”, “surgical or conservative management of the underlying disease”, “oral WHO1 pain medication” differed significantly between the two survey periods and were related to the results of the questions “How bearable was the pain during the last 24 hours?” and “Did you vomit during your ICU stay?”.

In both cases the only factor with significant influence on the changes from the first to the second period was the survey period itself (to “How bearable was the pain during the first 24 hours?” p = 0.001; to “Did you vomit during your ICU stay?” p = 0.026), while the other three factors showed no influence.

### Physicians

In the physician collective, no differences were found between the two survey periods regarding age, gender and working place.

Comparing both surveys, a similar share of participants knew about the existence of multidisciplinary work groups for pain management, about SOPs about responsibilities in pain management, SOPs for pharmacological treatment of problematic pain and constipation, SOPs for sedation (> 75% of participants for each item) and a SOP for non-pharmacological pain management at their ward (63.1% in 2012 and 70.8% in 2015). Although in 2015 on average more participants stated they were familiar with the respective standards, never was 100% and in only 2 of the 7 items (“At your ward, is there a written consent about the responsibilities in pain management?” and “Are written standards available that you can access when you face a problematic pain situation in a patient?”) >90% awareness was achieved (S1 Table, S1 Fig).

**Fig 1:**
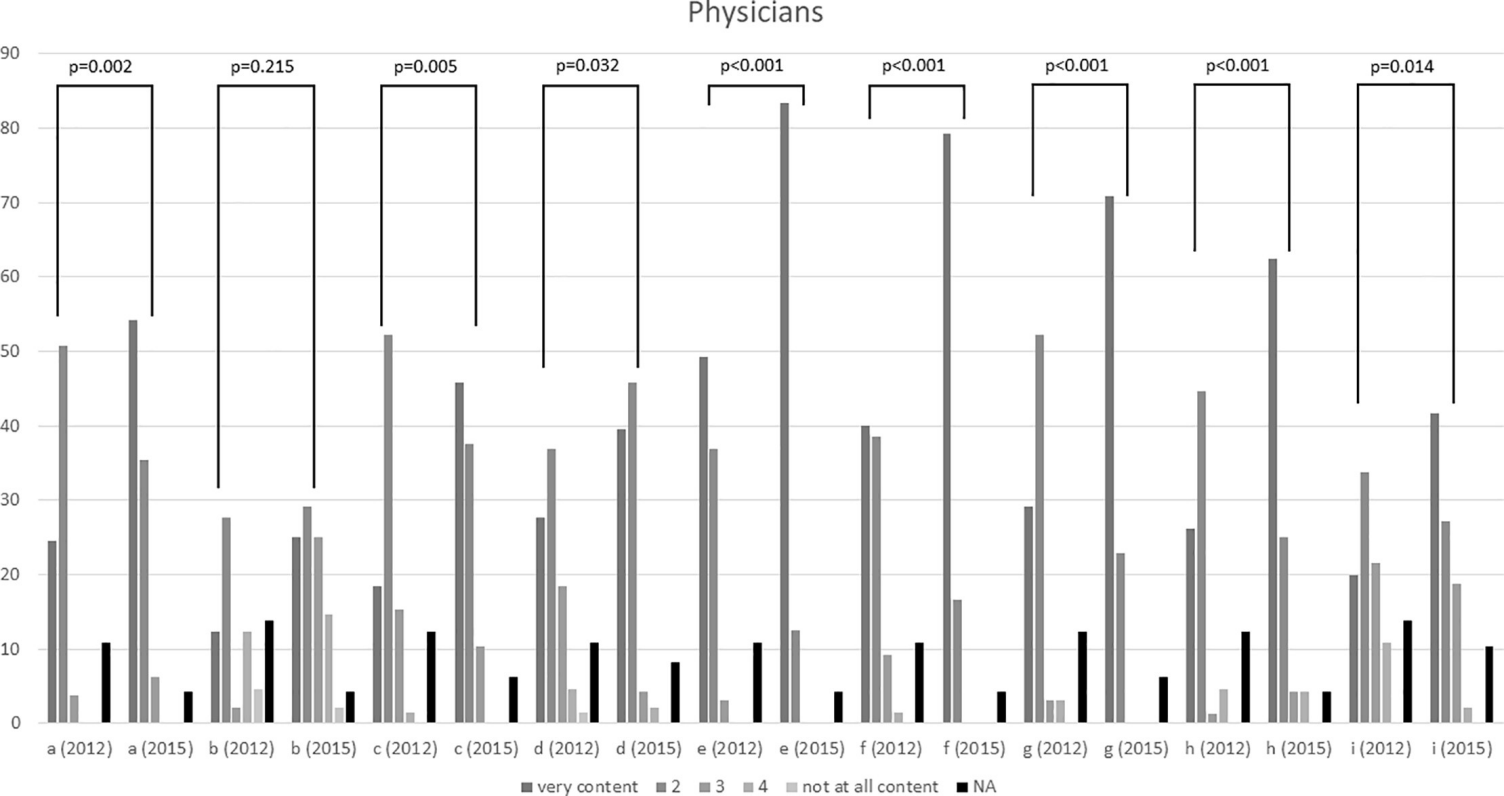
Physicians’ answers on question 12 a–i; comparison between 2012 and 2015. Values are absolute values, NA = not available.

Physicians from 2015 were more satisfied with their own (p = 0.002), with the nursing staff’s (p = 0.005) and the physiotherapists’ (p = 0.032) knowledge about pain medication. In addition, physicians’ satisfaction with communication between nurses and physicians (p<0.001), communication between physicians (p<0.001), communication between physiotherapists and nurses (p<0.001) as well as communication between physiotherapists and physicians (p = 0.001) improved significantly from 2012 to 2015. Furthermore, the 2015 collective was more pleased with pain management training offered at the department (p = 0.014). No differences were observed regarding the knowledge about non-pharmacological pain management options (Fig 1).

### Nurses

In the nursing staff collective, no differences were observed between the two surveys regarding age or gender. However, the 2015 collective reported longer working experience (p = 0.026).

**Fig 2:**
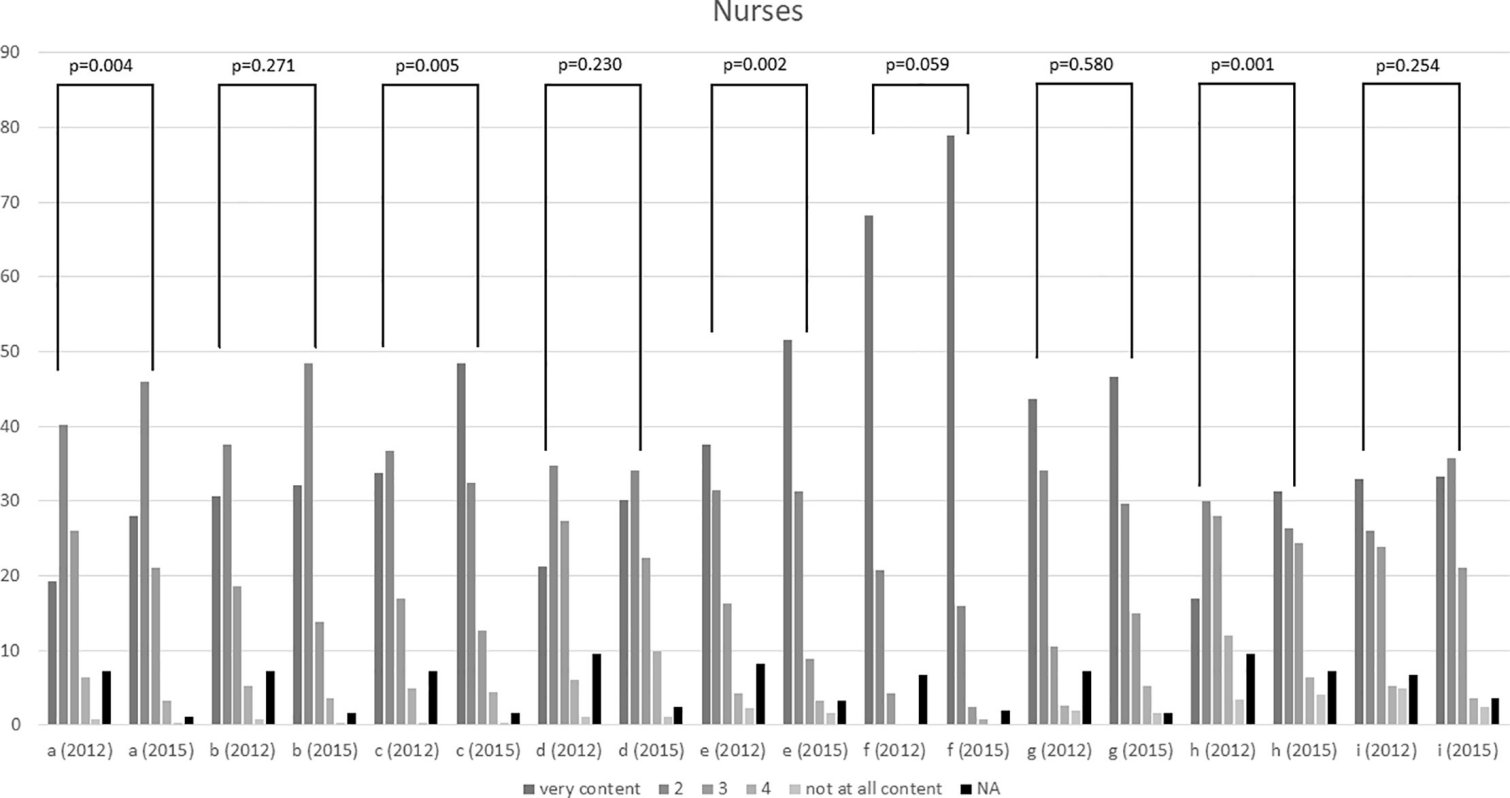
Nurses’ answers on question 12 a–i; comparison between 2012 and 2015. Values are absolute values, NA = not available.

Regarding questions concerning practices on the ward, more 2015 participants knew about the written standard for the treatment of constipation (p = 0.001). In 2015, also more participants stated to use transfer protocols which include the patient’s pain management (p = 0.031). Participants from the second survey were more satisfied with their own knowledge about pain medication (p = 0.004), the physician’s knowledge (p = 0.005), with communication between nurses and physicians (p = 0.002) and communication between physiotherapists and physicians (p = 0.001). No other differences were observed (S2 Table, S2 Fig, Fig 2).

Since the 2015 collective reported significantly longer working experience, linear regression models were performed relating the parameters “survey period” and “years of working experience” to the items of the questionnaire that changed between the two survey periods.

Awareness of the written standard for treatment of constipation correlated with both, the second survey period (p = 0.004) and the longer working experience (p<0.001). Satisfaction with the own knowledge of pharmacological pain management or the communication between nurses and physicians was associated with the 2015 collective (P = 0.016 and P = 0.016, respectively) and longer working experience (P = 0.001 and P<0.001, respectively). The results of the questions “Do you use transfer protocols that take the patient’s individual pain management into account?”, “How content are you with the physicians’ knowledge?”, and “How content are you with communication between therapists and physicians?” were only influenced by the survey period (P = 0.049, P = 0.029 and P = 0.003, respectively).

## Discussion

This is the first study reporting about the evaluation of the efficacy and medium-term sustainability of a pain PMQP implemented at nine ICUs of a University hospital. Patients, physicians and nurses from the hospital of the Medical University of Graz participated in two consecutive surveys. From the first survey carried out immediately after implementation of PMQP to the second survey three years thereafter, none of the evaluated parameters worsened, whilst the majority remained stable, and some even improved.

Overall, further PMQP exists and amongst those the benchmark project “QUIPS” is to improve the postoperative pain therapy through regular data collection, their analyses and feedback to clinics involved [16]. However, the focus of QUIPS is on the quality of results solely from the patients’ perspective, whereas our PMQP also focus on assurance of all quality aspects concerning the three dimensions of structure, process and outcome parameters incorporating all stakeholders of the PMQP, namely patients, physicians and nurses. Through this, continuous improvement based on 360 degree feedback is an essential part of our quality management process.

During both surveys, the median NRS scores for the most and least severe pain experienced since admission were 5 and 0 (of 10), respectively. Also in the linear regression analysis, no significant difference could be found between the two collectives. Gerbershagen and colleagues determined an NRS score of ≤4 as cut-off point between mild and moderate pain [17]. According to this benchmark, the patient-reported median NRS scores for most and least severe perceptions can be considered as “moderate pain” and “no pain”. These values appear especially low when considering that 50% of patients interviewed after ICU stay commonly rate their average pain levels as “moderate” to “severe” [18]. In a prospective cohort study, Desbiens et al. found that among critically ill nearly 15% were dissatisfied with pain control [19]. In comparison, the overall satisfaction with pain treatment was high among both of our collectives, reflected in a median score of 10 out of 10 in both cases with overall less than 10% reporting lower than average satisfaction with analgesic treatment. The subjective tolerance of pain levels experienced within 24 hours preceding the survey was overall very good. Patients questioned in 2015 deemed pain levels better bearable than those from the first survey in 2012. Regarding medicinal pain treatment, we found that during the second study period there was a tendency towards the administration of oral non-opioids. This change in pharmacotherapy was however within the wiggle room of guidelines and not due to general changes in the pain management protocol.

According to data from the literature, postoperative nausea and vomiting is considered by most patients as more distressing than postoperative pain [20]. What is more, vomiting may lead to serious complications such as wound dehiscence, dehydration or electrolyte imbalance [21]. Of all side effects assessed, vomiting occurred less often in patients of the second survey. Neither the lower rate of surgical patients, nor the increased requirement of artificial ventilation in the second survey influenced this result. The more frequent administration of oral non-opioids in the 2015 collective was also no confounder. Antiemetic treatment followed the same strict regimen in both periods and was given on demand or when deemed suitable. In contrast to our findings, Lehmkuhl et al. could not detect any differences in the incidence of postoperative nausea or vomiting after the implementation of an S3-guideline conform PMQP [7]. Still it is conceivable that the improvements in our study were indeed due to solidification of our PMQP, and that caregivers learnt to pay more attention not only to pain management, but also to side effects.

The surveys among the staff showed that both nurses and physicians were by large familiar with PMQP. From 2012 to 2015, participants from both professions reported increased satisfaction with both, intra- and inter-professional communication. Self-perception of medical knowledge, but also appreciation of the knowledge of partners from other professions increased from the first to the second survey. However, this self-perception was only in parts reflected by an increase of the staff’s actual knowledge of the implemented written standards and processes. In fact, knowledge of only one aspect (using structured transfer protocols that include the patient’s pain management) in the nursing staff collective improved, while in the physician collective familiarity with PMQP standards and processes remained fairly stable. Discrepancies between the health care personnel’s self-perception and actual medical knowledge have been described before: In a cross-sectional study amongst medical students, residents and appointed physicians, Aquirre-Raya et al. found that the de-facto familiarity with the cornerstones of evidence-based medicine was lower than estimated by the participants [22]. What is more, Andersson et al. reported that the implementation of evidence-based guidelines for pain management at an intensive care unit primarily changed the scope of prescribed pain medication from weak to strong opioids; at the same time, however, patients’ pain levels at rest and during movement remained the same [23]. Additionally the average years of working experience of the nursing staff had increased from 2012 to 2015; however, this could not be correlated to the given answers of the questionnaire.

Our PMQP may indeed have helped to solidify quality of pain management in everyday clinical practice. The biggest impact of PMQP seemingly was its strengthening effect on the employees’ self-efficacy concerning their own and their colleagues’ knowledge of pain management as well as the improved communication. These developments may have had beneficial effects on the patient’s wellbeing. This finding is of great importance regarding the effectiveness of the PMQP in a university hospital. It is well known that the turnover rate in university hospitals is higher than in smaller hospital settings as we have the major aim of educating medical and nursing students. Therefore, keeping up the knowledge at a high level over three years is an indicator of well-implemented PMQP.

A limitation of the study was the lack of a baseline-evaluation carried out before implementation of PMQP which probably remains the biggest limitation of the study. In addition, the two collectives were not entirely identical regarding the management of their underlying diseases, the presence of artificial ventilation and analgesic treatment. Furthermore, some parameters that further specify the patients’ characteristics, like e.g. length of stay at the ICU was not assessed. Yet another limitation was that the questionnaires used in this study–due to the fact that it was a pilot study–have not been validated beforehand. Since data in the three interrogated groups (patients, nurses, physicians) was collected independently no statistical link could be made between the groups. The data from the abovementioned audits proved to be too diverse to be statistically analysed. Hence this data was excluded from the study. Nevertheless, with the present study design the sustainability of the program could be evaluated.

## Conclusions

In summary, although management and treatment of patients need to be individualized and depends on the clinical experience of the attending staff, a PMQP as ours described above helps to focus on high quality care based on Evidence-Based Medicine. Since the minor changes observed in pharmacological treatment and in patient characteristics did not influence the improvements in outcome we observed between the two periods, it does not seem too far-fetched to conclude that these improvements were due to continuous use of the guidelines. Continuous education, ongoing training, regular updates and implementation of feedback-loops ensure continuity, in some parameters even an increase in knowledge and competencies. This is mirrored in high patient and staff member satisfaction. Our pilot study demonstrated that even intensive care units may benefit from the implementation of PMQPs, as in our case in pain management. Further studies with higher number of participants, validated questionnaires, pre- and post-implementation assessments, and long-term evaluation needs to confirm our results.

## Supporting information

S1 Table Survey results of physicians. (DOCX)

S2 Table Survey results of nurses. (DOCX)

S1 Fig Scheme for sedation and analgesia at ICUs (original document). (DOCX)

S2 Fig Treatment regimen for constipation at ICUs (translated version). (DOCX)
