# Supplementary material for: Implementation and maintenance of a pain management quality assurance program at intensive care units: 360 degree feedback of physicians, nurses and patients
Source: PLoS One. 2018 Dec 19;13(12):e0208527. doi: 10.1371/journal.pone.0208527 (PMC6300320; doi:10.1371/journal.pone.0208527)
Supplement: S1 Table — (DOCX) [file pone.0208527.s001.docx]

**S1 Table:** Survey results of physicians. Dnk = Do not know

| **Parameter (physicians)** | **Physicians, n (%)** | **Characteristics 2012** | **Characteristics 2015** | **Test, P-value** |
| --- | --- | --- | --- | --- |
| *Gender* | Male: 52 (46.0%)  Female: 53 (46.9%)  Missing: 8 (7.1%) | Male: 28 (43.1%)  Female: 30 (46.2%)  Missing: 7 (10.7%) | Male: 24 (50.0%)  Female: 23 (47.9%)  Missing: 1 (2.1%) | Fisher’s test, P=0.845 |
| *Total (n)* | 113 | 65 | 48 | - |
| *Age (years)* | <40: 34 (30.1%)  40-49: 48 (42.5%)  50-60: 22 (19.5%)  >60: 1 (0.8%)  Missing: 8 (7.1%) | <40: 20 (30%)  40-49: 28 (43.1%)  50-60: 9 (13.8%)  >60: 1 (1.5%)  Missing: 7 (10.7%) | <40: 14 (29.2%)  40-49: 20 (41.7%)  50-60: 13 (27.0%)  >60: 0  Missing: 1 (2.1%) | Spearman-correlation, P=0.335 |
| *Surgical (S) or conservative (C) ICU* | S: 73 (64.6%)  C: 40 (35.4%) | S: 41 (63.1%)  C: 24 (36.9%) | S: 32 (66.7%)  C: 16 (33.3%) | Fisher’s test, P=0.842 |
| *1. Multidisciplinary work group for pain management?* | Yes: 96 (85.0%)  No: 2 (1.7%)  Dnk: 15 (13.3%) | Yes: 56 (86.2%)  No: 0  Dnk: 9 (13.8%) | Yes: 40 (83.4%)  No: 2 (4.2%)  Dnk: 6 (12.4%) | Fisher’s test,  P=0.181 |
| *2. Written consent responsibilities pain management?* | Yes: 97 (85.8%)  No: 3 (2.7%)  Dnk: 13 (11.5%) | Yes: 53 (81.5%)  No: 1 (1.5%)  Dnk: 11 (17.0%) | Yes: 44 (91.6%)  No: 2 (4.2%)  Dnk: 2 (4.2%) | Fisher’s test,  P=0.593 |
| *3. Written standards for problematic pain situation?* | Yes: 100 (88.5%)  No: 2 (1.7%)  Dnk: 11 (9.8%) | Yes: 55 (84.7%)  No: 1 (1.5%)  Dnk: 9 (13.8%) | Yes: 45 (93.7%)  No: 1 (2.1%)  Dnk: 2 (4.2%) | Fisher’s test, P=1.000 |
| *4. Written standard for treatment of nausea/vomiting?* | Yes: 93 (82.2%)  No: 9 (8.0%)  Dnk: 11 (9.8%) | Yes: 53 (81.5%)  No: 5 (7.8%)  Dnk: 7 (10.7%) | Yes: 40 (83.4%)  No: 4 (8.3%)  Dnk: 4 (8.3%) | Fisher’s test, P=1.000 |
| *5. Written standard for treatment of constipation?* | Yes: 95 (84.1%)  No: 7 (6.2%)  Dnk: 11 (9.8%) | Yes: 53 (81.5%)  No: 4 (6.2%)  Dnk: 8 (12.3%) | Yes: 42 (87.5%)  No: 3 (6.25%)  Dnk: 3 (6.25%) | Fisher’s test, P=1.000 |
| *6. Written standard for sedation?* | Yes: 93 (82.2%)  No: 5 (4.5%)  Dnk: 15 (13.3%) | Yes: 51 (78.5%)  No: 3 (4.6%)  Dnk: 11 (17.0%) | Yes: 42 (87.5%)  No: 2 (4.2%)  Dnk: 4 (8.3%) | Fisher’s test, P=1.000 |
| *7. Written standard for non-pharmacological pain management?* | Yes: 75 (66.4%)  No: 9 (8.0%)  Dnk: 29 (25.6%) | Yes: 41 (63.1%)  No: 4 (6.2%)  Dnk: 20 (30.7%) | Yes: 34 (70.8%)  No: 5 (10.4%)  Dnk: 9 (18.8%) | Fisher’s test, P=0.727 |
