# Supplementary material for: Implementation and maintenance of a pain management quality assurance program at intensive care units: 360 degree feedback of physicians, nurses and patients
Source: PLoS One. 2018 Dec 19;13(12):e0208527. doi: 10.1371/journal.pone.0208527 (PMC6300320; doi:10.1371/journal.pone.0208527)
Supplement: S2 Table — (DOCX) [file pone.0208527.s002.docx]

**S2 Table:** Survey results of nurses. Dnk = Do not know

| **Parameter (nurses)** | **Nurses, n (%)** | **Characteristics 2012** | **Characteristics 2015** | **Test, P-value** |
| --- | --- | --- | --- | --- |
| *Gender* | Male: 94 (18.4%)  Female: 404 (79.2%)  Missing: 12 (2.4%) | Male: 41 (15.5%)  Female: 212 (80.3%)  Missing: 11 (4.2%) | Male: 53 (21.5%)  Female: 192 (78.0%)  Missing: 1 (0.5%) | Fisher’s test, P=0.137 |
| *Age (years)* | <30: 226 (44.3%)  30-39: 162 (31.7%)  40-50: 81 (15.9%)  >50: 32 (6.3%)  Missing: 9 (2.4%) | <30: 121 (45.8%)  30-39: 82 (31.1%)  40-49: 39 (14.8%)  >50: 14 (5.3%)  Missing: 8 (3.0%) | <30: 105 (42.7%)  30-39: 80 (32.5%)  40-49: 42 (17.1%)  >50: 18 (7.3%)  Missing: 1 (0.4%) | Spearman-correlation, P=0.239 |
| *Years of working experience in intensive care* | <2: 119 (23.3%)  3-5: 101 (19.8%)  5-10: 114 (22.4%)  >10: 160 (31.4%)  Missing: 16 (3.1%) | <2: 75 (28.4%)  3-5: 50 (19.0%)  5-10: 45 (17.0%)  >10: 79 (29.9%)  Missing: 15 (5.7%) | <2: 44 (17.9%)  3-5: 51 (20.7%)  5-10: 69 (28.1%)  >10: 81 (32.9%)  Missing: 1 (0.4%) | Spearman-correlation,  P=0.026 |
| *Main group of patients treated; surgical (S), conservative (C), mixed (M)* | S: 282 (55.3%)  C: 182 (35.6%)  M: 35 (6.9%)  Missing: 11 (2.2%) | S: 149 (56.4%)  C: 87 (33.0%)  M: 18 (6.8%)  Missing: 10 (3.8%) | S: 133 (54.1%)  C: 95 (38.6%)  M: 17 (6.9%)  Missing: 1 (0.4%) | Chi², P=0.569 |
| *1. Multidisciplinary work group for pain management?* | Yes: 474 (92.9%)  No: 11 (2.2%)  Dnk: 25 (4.9%) | Yes: 242 (91.7%)  No: 6 (2.3%)  Dnk: 16 (6.0%) | Yes: 232 (94.3%)  No: 5 (2.0%)  Dnk: 9 (3.7%) | Fisher’s test,  P=1.000 |
| *2. Written consent responsibilities pain management?* | Yes: 472 (92.5%)  No: 12 (2.4%)  Dnk: 26 (5.1%) | Yes: 237 (89.8%)  No: 6 (2.3%)  Dnk: 21 (7.9%) | Yes: 235 (95.5%)  No: 6 (2.5%)  Dnk: 5 (2.0%) | Fisher’s test,  P=1.000 |
| *3. Written standards for pharmacological pain treatment?* | Yes: 402 (78.8%)  No: 75 (14.7%)  Dnk: 33 (6.5%) | Yes: 199 (75.4%)  No: 41 (15.5%)  Dnk: 24 (9.1%) | Yes: 203 (82.5%)  No: 34 (13.8%)  Dnk: 9 (3.7%) | Fisher’s test, P=0.451 |
| *4. Written standard for treatment of nausea/vomiting?* | Yes: 265 (52.0%)  No: 189 (37.0%)  Dnk: 56 (11.0%) | Yes: 129 (48.9%)  No: 104 (39.4%)  Dnk: 31 (11.7%) | Yes: 136 (55.3%)  No: 85 (34.6%)  Dnk: 25 (10.1%) | Fisher’s test, P=0.184 |
| *5. Written standard for treatment of constipation?* | Yes: 348 (68.2%)  No: 130 (25.5%)  Dnk: 32 (6.3%) | Yes: 160 (60.6%)  No: 82 (31.1%)  Dnk: 22 (8.3%) | Yes: 188 (76.4%)  No: 48 (19.5%)  Dnk: 10 (4.1%) | Fisher’s test, P=0.001 |
| *6. Written standard for sedation?* | Yes: 280 (54.9%)  No: 177 (34.7%)  Dnk: 53 (10.4%) | Yes: 133 (50.4%)  No: 95 (36.0%)  Dnk: 36 (13.6%) | Yes: 147 (59.8%)  No: 82 (33.3%)  Dnk: 17 (6.9%) | Fisher’s test, P=0.213 |
| *7. Written standard for non-pharmacological pain management?* | Yes: 347 (68.0%)  No: 103 (20.2%)  Dnk: 60 (11.8%) | Yes: 165 (62.5%)  No: 58 (22.0%)  Dnk: 41 (15.5%) | Yes: 182 (74.0%)  No: 45 (18.3%)  Dnk: 19 (7.7%) | Fisher’s test, P=0.144 |
| *8. Written standard for preventive pharmacological pain therapy before nursing measures?* | Yes: 277 (54.3%)  No: 159 (31.2%)  Dnk: 74 (14.5%) | Yes: 133 (50.4%)  No: 87 (33.0%)  Dnk: 44 (16.6%) | Yes: 144 (58.5%)  No: 72 (29.3%)  Dnk: 30 (12.2%) | Fisher’s test, P=0.196 |
| *9. When you call a physician for a drug prescription, how long do you have to wait for it?* | ≤15 min: 471 (92.4%)  >15 min: 16 (3.1%)  >30 min: 6 (1.2%)  >60 min: 1 (0.2%)  Missing: 16 (3.1%) | ≤15 min: 233 (88.3%)  >15 min: 9 (3.4%)  >30 min: 5 (1.9%)  >60 min: 1 (0.4%)  Missing: 16 (6.0%) | ≤15 min: 238 (96.7%)  >15 min: 7 (2.9%)  >30 min: 1 (0.4%)  >60 min: 0  Missing: 0 | Spearman-correlation, P=0.133 |
| *10. Do you attend pain management training at least once a year?* | Yes: 138 (27.1%)  No: 343 (67.2%)  Missing: 29 (5.7%) | Yes: 66 (25.0%)  No: 171 (64.8%)  Missing: 27 (10.2%) | Yes: 72 (29.3%)  No: 172 (69.9%)  Missing: 2 (0.8%) | Fisher’s test, P=0.762 |
| *11. Do you use transfer protocols that take the patient’s pain management into account?* | Yes: 292 (57.3%)  No: 161 (31.6%)  Missing: 29 (5.7%) | Yes: 130 (49.2%)  No: 89 (33.8%)  Missing: 45 (17.0%) | Yes: 162 (65.9%)  No: 72 (29.3%)  Missing: 12 (4.8%) | Fisher’s test, P=0.031 |
