# Supplementary material for: Implementation and maintenance of a pain management quality assurance program at intensive care units: 360 degree feedback of physicians, nurses and patients
Source: PLoS One. 2018 Dec 19;13(12):e0208527. doi: 10.1371/journal.pone.0208527 (PMC6300320; doi:10.1371/journal.pone.0208527)
Supplement: S1 Fig — (DOCX) [file pone.0208527.s003.docx]

| **S1 Fig:** Scheme for sedation and analgesia at ICUs   Medikamentöses Behandlungsschema ICU 2/3 /4 | | |  |  |
| --- | --- | --- | --- | --- |
| Die hier vorliegenden Beispiele sind als Anregung gedacht. Sie sind nicht bindend und nicht haftungsbefreiend. Alle Angaben beziehen sich auf Tagesdosen. Kontraindikationen, Medikamentenkombinationen und Wechselwirkungen von Arzneimitteln können in dieser Schemaform nicht berücksichtig werden. Die medikamentöse Therapie muss immer individuell für jeden Patienten vom Arzt verordnet werden.  Indikationen:  1 Postoperatives Weaning beim unkomplizierten Verlauf  2a Intensivbehandlung: Eskalationsstufe (beim erhöhten Sedierungsbedarf)  2b Intensivbehandlung: schweres Schädel-Hirn-Trauma  3 Extubierte Patienten  4 Delir | | |  |  |
|  |  |  |  |  |
| Patienten-gruppe | Sedativum  SAS 🡪 Zielvorgabe bei der Intensivvisite | Analgetika  BPS, NRS, FPS 🡪 Zielvorgabe max. 3-5 |  |  |
|  | | |  |  |
| 1 | Propofol 1 - 3 mg/kg/h i.v  (möglichst bald reduzieren) | Paracetamol 1 g 3 x 1 i.v., oder Novalgin 1 g/KI 3 x 1 i.v. oder  Diclofenac 75 mg/KI 2 x 1 i.v. |  |  |
|  |  | Additiv für Analgesie und Tubustoleranz:  Remifentanil initial 0,02 – 0,3 µg/kg/min i.v. ,  dann reduzieren auf die kleinste klinisch sinnvolle Dosis |  |  |
|  | | |  |  |

|  | | |
| --- | --- | --- |
| **2a** | **Propofol 1 - 3 mg/kg/h i.v**  **Additiv bei erhöhtem Sedierungsbedarf:**  **S-Ketamin 25 – 300 mg/h i.v**  **Alternativ / additiv beim agitierten Patienten  in eskalierender Reihenfolge:**  **Praxiten 15 mg 3 x 1 p.o.** (bis 50mg 6 x 1 p.o.), und/oder  **Seroquel 25 mg 2 x 1 p.o.** (bis 100mg 3x1 p.o.)**,** oder  **Haldol** **2,5 mg/KI 1 x 1 i.v** (bis 5mg/KI 1x1 i.v.).,  **Additiv frühzeitig bei speziellen Indikationen:**  **Venlafaxin 75 mg ret. 1-0-0 p.o.,** oder  **Mirtazapin 15 mg 0-0-1 p.o.** | **Siehe Stufe 1** |
|  | | |
| **2b** | **Propofol 1 - 3 mg/kg/h i.v**  **Additiv bei erhöhtem Sedierungsbedarf:**  **S-Ketamin 25 – 300 mg/h i.v**  **Additiv:** Midazolam 10 – 20 mg/h i.v. **Additiv zur Burst Suppression:**  **Brietal 50 – 300 mg/h i.v. (Alternativ Thiopental)** | **Für Analgesie und Tubustoleranz:**  **Sufentanil initial 0,1 – 0,2 mg/h i.v., dann reduzieren auf die kleinste klinisch sinnvolle Dosis** |
|  | | |

|  | | |
| --- | --- | --- |
| **3** | **Praxiten ausschleichen**  **Additiv bei agitierten Patienten:**  **Seroquel 25 mg 2 x 1 p.o.**  **Additiv frühzeitig bei speziellen Indikationen**  **Venlafaxin 75 mg ret. 1-0-0 p.o.,** oder  **Mirtazapin 15 mg 0-0-1 p.o.** | **Paracetamol 1 g 3 x 1 i.v.**  **Alternativ bzw. bei speziellen Indikationen:**  **Novalgin 4 x 30 gtt (oder 1 g/KI 3 x 1 i.v. ),** oder  **Diclofenac 50 mg max. 3 x 1 p.o. (oder 75 mg/KI 2 x 1 i.v.)**  **Additiv:**  **Targin 10/5 mg p.o. max. 4 x 1 p.o.,** oder **Dipidolor 3mg repetitiv i.v.;** Dosierungsintervall mind. 10 min, max. 5 Boli / Stunde; max. 30mg / 24 Stunden **Bei Überschreiten der Dosierungsgrenzen 🡪 Schmerzen bzw. Therapie neu evaluieren!** **🡪** letzte Opioidgabe ca. 60 min vor Entlassung  **Bei Kurzeingriffen:**  **Effentora 100 µg s.l.,** und/oder  **S-Ketamin 15 – 50 mg i.v. als Bolus** |
|  | | |
| **4** | **Haldol** **2,5 mg/KI 1 x 1 i.v** (bis 5mg/KI 1x1 i.v.)., oder  **Seroquel 25 mg 2 x 1 p.o.** (bis 100mg 3x1 p.o.)**,**  **Additiv:**  **Praxiten 15 mg 3 x 1 p.o.** (bis 50mg 6 x 1 p.o.), und/oder  **Bevitol 500 mg/KI 1x1 i.v**. (bei Alkohol-Abusus oder bei unbekanntem Koma wegen suspektem Vit. B1-Mangel) |  |
|  | | |

| **Therapie von Übelkeit und Erbrechen:**    **Stufe I: Paspertin 10mg,** oder  **Ondansetron**  3 x 4 mg i.v.  **Stufe II: PONVeridol** 1,25 mg i.v.  **Stufe III: Dexamethason** 8 mg i.v. |
| --- |
| **Therapie von Atemnot:**  **Naloxon: 0,08 – 0,2 mg repetitiv i.v.** (max. 0,8 mg, 1 Amp. = 0,4 mg ad 10 ml NaCl 0,9% 🡪 davon 2 – 5 ml) |
| **Therapie der Obstipation:**  **Vorgehen nach Stationsstandard bzw. postoperativ in Absprache mit dem behandelnden Chirurgen** |
| **Therapie von Juckreiz:**  **Fenistil 4 mg: max. 2 x 1 i.v (für max. 7 Tage),** oder  **Fenistil Gel: max. 4 x tgl. lokal,** oder  **Zyrtec 10 mg: 1 x 1 p.o.** |

**Legende:**

| **Abkürzung** | **Begriff** |
| --- | --- |
| Amp. | Ampullen |
| BPS | Behavioral Pain Scale |
| FPS | Faces Pain Scale |
| i.v. | intravenös |
| /KI | als Kurzinfusion |
| KI: | Kontraindikation |
| max. | maximal |
| NaCl | Kochsalzlösung |
| NRS | Numeric Rating Score |
| p.o. | per os |
| SAS | Sedation-Agitation Score |
| s.l. | sublinqual |
| tgl. | täglich |

|  | Name | Datum | Unterschrift |
| --- | --- | --- | --- |
| Erstellt von: | Univ.Prof.Dr. A. Sandner-Kiesling | 27.07.2015 |  |
| Geprüft von: | Univ.Prof.DDr. P. Metnitz | 27.07.2015 |  |
| Freigegeben von: | Univ.Prof.DDr. P. Metnitz | 27.07.2015 |  |

**Übersicht der im Schema verwendeten Medikamente**

(Die relative Opioid-Wirkstärke bezieht sich auf Morphin = 1)

| **Wirkstoff/Handelsname** | **Wirkung/Eigenschaften** | **Nebenwirkung/ Kontraindikation** |
| --- | --- | --- |
| **Buprenorphin / Transtec® Temgesic®** | Opioid/ partieller Agonist µ-Rezeptor und  Antagonist am κ-Rezeptor, Natriumkanalblocker   wirkt antineuropathisch  Wirkdauer TDS 24-96 Stunden; Wechsel des  transdermalen Pflasters alle 4 Tage  Relative Wirkstärke: **100** | siehe Piritramid jedoch weniger atemdepressiv als reine  µ-Rezeptor Agonisten, Ausscheidung rein hepatisch (ideal bei NINS)!  Wirkeintritt des transdermalen Pflasters nach 24h!  Transtec**®**: 35 bzw. 52,5 bzw. 70 µg/h  Rescue Medication: **Temgesic**® 0,2 - 0,4 mg (Wirkeintritt nach 45 min,   Wirkdauer 8h) |
| **Cetirizin / Zyrtec®** | Antihistaminikum, Antiemetikum | **CAVE:** Überempfindlichkeit  **KI:** NINS, Epilepsie, Krampfneigung |
| **Dexamethason / Fortecortin®** | Kortikoid  Antiödematös, antiemetisch, antineuropathisch,   antiphlogistisch | **CAVE:** Überempfindlichkeit, abruptes Absetzen  schwer einstellbarer Blutdruck, Diabetes mellitus, GI-Ulzera,,  Osteoporose, kardiale Insuffizienz, Glaukom, Suizidneigung  **KI:** systemische Infektionen |
| **Dimentiden / Fenistil®** | Antihistaminikum, Antiemetikum | **CAVE:** Müdigkeit, Mundtrockenheit oder Hitzegefühl. Gelegentlich   gastrointestinales Unbehagen  **KI:** Überempfindlichkeit gegen den Wirkstoff oder einen der sonstigen   Bestandteile, Einnahme von MAO-Hemmern, Kinder bis 12 Jahre. |
| **Droperidol / Ponveridol®** | Neuroleptikum, Antiemetikum | **CAVE:** Komedikation mit anderen Neuroleptika, Epilepsie, Hypovolämie,   malignes neuroleptisches Symptom  **KI:** Überempfindlichkeit, bekanntes oder vermutetes verlängertes   QTc-Intervall , Hypokaliämie oder Hypomagnesiämie, Bradykardie,   Phäochromozytom, komatöse Zuständen, Parkinson-Krankheit, schwere   Depression |
| **Diclofenac / Voltaren®** | Diclofenac: analgetisch, antiphlogistisch,  antipyretisch  Wirkung: schwache COX I-, starke COX II-Hemmung  Metabolisierung in der Leber | **CAVE** vermehrte cerebro-/kardiovaskuläre Ereignisse unter Diclofenac  **KI:** Allergie auf NSAIDS, GFR < 50 ml/min/1,73m^2^,   Ulcus(-anamnese), Leberinsuffizienz, Epilepsie, relativ: KHK, PAVK,   Herzinsuffizienz, arterielle Hypertonie |
| **Fentanyl / Lafene® Effentora®** | Opioid/ µ-Rezeptor Agonist  wirkt **NICHT** antineuropathisch  Glukuronisierung in d. Leber, inaktive Metaboliten  Wirkbeginn (i.v.) : < 2-3 min, Wirkdauer: 20-30 min;   Relative Wirkstärke: **70- 100** | siehe Piritramid  sowie Husten, Juckreiz;   **CAVE** bei NINS 🡪 Kumulation! |
| **Haloperidol/Haldol®** | Neuroleptikum  Antipsychotisch  TMD 40 - 60 mg | **CAVE:** als Kurzinfusion nur in Glucose 5% oder Mannit lösbar i.v..   In NaCl gelöst ist ein Ausflocken möglich!!   **CAVE:** i.v. = Off-Label Use !!)  **KI:** Koma, ZNS-Depression, M. Parkinson  **NW:** Extrapyramidale Symptome, tardive Dyskinesie, malignes  neuroleptisches Syndrom, ZNS, Magen/Darm, Endokrinium,   Herz/Kreislauf, Augen, Leber, Urogenitale |
| **Hydromorphon / Hydal®** | Opioid/ µ-Rezeptor Agonist  wirkt **NICHT** antineuropathisch  Vorteil: niedrige Plasmaeiweißbindung,  Metabolisierung in der Leber,   Wirkdauer retardiert 8-12h, unretartiert 3-4 Std  Relative Wirkstärke: **5-7,5** | siehe Piritramid  gut einsetzbar bei NINS, da keine aktive Metaboliten; |
| **Ketamin / Ketanaest-S®** | i.v. Anästhestikum | **CAVE:** bei Herzinsuffizienz und unbehandelter Hypertonie, instabiler   Angina pectoris, gesteigertem Hirndruck, Glaukom, perforierenden   Augenverletzungen, Alkoholeinfluss, Patienten mit einer schweren   psychischen Störungen, bei unzureichend behandelter Hyperthyreose  **KI:** Risiko für Hypertonie oder gesteigerten Hirndruck, alleiniges   Anästhetikum bei manifesten ischämischen Herzerkrankungen,   Präeklampsie, Eklampsie. |
| **Metohexital / Brietal®** | i.v. Anästhestikum (Barbiturat) | **CAVE**: Muskelzuckungen, Laryngospasmus, Schluckauf, Husten, Hypotonie,  Thrombophlebitis, lokale Schmerzen, Atemdepression, Bronchospasmus,  ZNS, Magen, Darm, Haut,  **KI**: Porphyrie, Status asthmaticus, akute Vergiftungen mit ZNS-Dämpfern |
| **Metamizol / Novalgin®** | Analgetische, antipyretische Wirkung,  spasmolytische Wirkung  Wirkmechanismus: nicht geklärt  Wirkdauer: ca. 2,5 - 4 h | **CAVE:** langsame Infusion > 30 min 🡪 RR- Abfall möglich,  Blutbild-Kontrollen  **KI:** Allergie, Leukopenie, schwere Leber- bzw. Nieren-  Funktionsstörung, Glucose-6-Phosphat-Dehydrogenasemangel,  Porphyrie |
| **Metoclopramid / Paspertin®** | Prokinetikum, Dopamin D2-Antagonist | **CAVE:** Müdigkeit, Schlaflosigkeit, Schwindel, Kopfschmerzen,   Depressionen, Durchfall, malignes neuroleptisches Syndrom,   extrapyramidale Störungen vor allem bei Kindern, bei Langzeitgabe   Spätdyskinesien, Parkinson-Syndrom; Gynäkomastie, Störungen der   Regelblutung, Hypotonie, Überempfindlichkeit  **KI:** Phäochromozytom, Epilepsie, Magen/Darm-Blutungen, -Obstruktionen,  Prolaktin-abhängige Tumore, M. Parkinson, Spätdyskinesien,   extrapyramidale Störungen, Methämoglobinämie durch Metoclopramid |
| **Mirtazapin** | Antidepressivum  wirkt antineuropathisch | **CAVE:** Epilepsie und hirnorganischem Psychosyndrom; Leber- oder   Niereninsuffizienz; Herzerkrankungen, QT-Verlängerung, Hypotonie,   Miktionsstörungen; akutem Engwinkelglaukom; Diabetes mellitus  **KI:** keine  **NW:** Appetit- und Gewichtszunahme, Schläfrigkeit, Sedierung,   Kopfschmerzen, trockener Mund, Gewichtszunahme, (orthostatische)   Hypotonie, Schwindel, Erschöpfung, Manie, Konvulsionen, Akathisie,   Tremor, Muskelzuckungen, Ödeme, Knochenmarksdepression,   vorübergehender Anstieg der Transaminasen, Exantheme, Parästhesie,   Arthralgie/Myalgie, Hyponatriämie, Albträume, Serotoninsyndrom |
| **Naloxon** | Opioidantagonist | **Targin = Oxycodon + Naloxon**  **CAVE:** Entzugssyndrom  **KI:** keine **NW:** ZNS, Allergien, Herz, Kreislauf, Magen/Darm, Haut, postoperative   Schmerzen, lokale Reizung |
| **Ondansetron / Zofran®** | Serotonin-Antagonist, Antiemetikum | **CAVE:** Schwangerschaft und Stillperiode  **KI:** keine  **NW:** Überempfindlichkeit, Kopfschmerzen, Flush, Schluckauf, Mundtrockenheit, lokale Reizung an der Einstichstelle, Obstipation, Sehstörungen (vorübergehende Blindheit), Leber, Vertigo, Krämpfe, extrapyramidale Reaktionen, Serotoninsyndrom, Brustschmerzen, QT-Verlängerung |
| **Oxacepam / Praxiten®** | Sedierendes Benzodiazepin  TMD 100 mg | **KI:** Myasthenie, Intoxikation mit Alkohol oder ZNS - Dämpfern,  schwere Ateminsuffizienz, Schlaf - Apnoe - Syndrom, schwere   Leberschäden  **NW:** Sedierung, ZNS, Depression, Konvulsionen, Magen/Darm,  Haut, Sehstörungen, paradoxe Reaktionen, anterograde   Amnesie, Blutbild, Überempfindlichkeit, Atemdepression |
| **Oxycodon / Oxygerolan®** | Opioid/ µ- und κ-Rezeptor Agonist;   wirkt antineuropathisch  Metabolisierung in der Leber mit 1 Hauptmetabolit,  Wirkdauer: retardiert 8 -12 h, unretardiert 3h,   Wirkstärke: **1,5 – 2** | **CAVE:** NINS, Leberinsuffizienz |
| **Paracetamol / Mexalen®** | Nichtopioid: analgetisch, antipyretisch  TMD: Mexalen 2000mg, Perfalgan 4000mg | **KI:** Allergie, Schwerer Leberschaden, Alkoholabusus  **NW:** Leberversagen |
| **Piritramid / Dipidolor®** | Opioid/ µ-Rezeptor Agonist  Metabolisierung in der Leber, keine Metaboliten  Wirkeintritt nach 2-3 min, Wirkdauer: 6-8 h;   Relative Wirkstärke: **0,7** | **CAVE:** Koma, Atemdämpfung, Porphyrie, Alkoholintoxikation  **NW:** Atemdepression, Übelkeit, Obstipation |
| **Propofol / Diprivan®** | kurzwirksames i.v. Anästhestikum | **CAVE:** PRIS (Maximaldosis: 4mg/kg/h), Überempfindlichkeit   gegen Soja oder Erdnuss,  **NW:** Blutdruckabfall, Atemdepression, Anaphylaxie, Psyche,  ZNS (Krämpfe), Hypertriglyzeridämie, Bradykardie, Arrhythmien,  Übelkeit, Erbrechen, Atmung, lokale Reaktion, postoperatives   Fieber |
| **Quetiapin / Seroquel®** | Atypisches Antipsychotikum mit antidepressiver  Komponenten  TMD 300 mg | **KI:** (keine Eintrag) **NW:** ZNS (Kopfschmerzen), Magen/Darm (Obstipation,   Darmverschluss), Dysphagie, Rhinitis, Gewichtszunahme,   QT-Verlängerung, orthostatische Hypotonie, Krampfanfälle,   extrapyramidale Symptome, Spätdyskinesie, malignes   neuroleptisches Syndrom, Leukopenie/Neutropenie,   Enzymanstiege, Anstieg von Triglyzeriden und   Gesamtcholesterol, Senkung des HDL-Cholesterols,   Hyperglykämie, Pankreatitis, Entzugssymptom |
| **Remifentanil/Ultiva®** | Opioid/ µ-Rezeptor Agonist  Hydrolyse durch Blut/Gewebeesterasen  Wirkdauer: 8 - 10 min;   Relative Wirkstärke: **75-120** | **siehe Piritramid  keine Bolusgaben!** |
| **Sufentanil / Sufenta®** | Opioid/ µ-Rezeptor Agonist  Metabolisierung vornehmlich hepatisch  Wirkdauer: 20-25 min ;   Relative Wirkstärke: **1000** | **siehe Piritramid** |
| **Venlafaxin / Efectin®** | Antidepressivum (SNRI)   wirkt antineuropathisch | **CAVE:**  **KI:** Schwere Hypertonie, Engwinkelglaukom, Prostataleiden, schwere   Leber-, Nierenschäden  **NW:** Herz (QT-Verlängerung, Arrhythmien), abnorme Blutungen, ZNS,   Auge, Tinnitus, Magen/Darm, Mundtrockenheit, Harndrang, Schwitzen,   Haut, erhöhte Cholesterinwerte, Kreislauf, Leber, erektile Dysfunktion,   Verschlechterung eines Diabetes, Serotoninsyndrom, Psyche (suizidales   Verhalten), Beeinflussung von Labortests, beim abrupten Absetzen   Entzugserscheinungen |
