# Supplementary material for: Implementation and maintenance of a pain management quality assurance program at intensive care units: 360 degree feedback of physicians, nurses and patients
Source: PLoS One. 2018 Dec 19;13(12):e0208527. doi: 10.1371/journal.pone.0208527 (PMC6300320; doi:10.1371/journal.pone.0208527)
Supplement: S2 Fig — (DOCX) [file pone.0208527.s004.docx]

**S2 Fig:** Standard for Treatment of Constipation in ICU patients

# Non-abdominal surgical patient (no intervention on the gastrointestinal tract), non-surgical patient

**3^rd^ postoperative day/3^rd^ day at ICU:** 1 or 2 enemas with foregut

If insufficient additionally 2 Dulcolax suppositories or 2 Microlax

**4^th^ postoperative day/4^th^ day at ICU:** 1 or 2 enemas with foregut, 2 Dulcolax suppositories

From **5^th^ postoperative day/5^th^ day at ICU** on: individual constipation treatment according to medical prescription

Measures:

- Neostigmine-perfusor: 2.5mg ad 50ml NaCl 0.9% IV once during 4 hours at 12.5ml/h or during 24 hours at 2ml/h
- Gastrografin: once 50-100ml via gastric tube or orally
- Methylnaltrexone: 12mg SC

Additional measures may be administered according to medical prescription from the 2^nd^ postoperative day/2^nd^ day at ICU:

- Prune juice: up to 200ml via gastric tube or orally
- Laevolac oral solution: 1 or 2 tablespoons 2 or 3 times daily via gastric tube or orally
- Guttalax: 10 to 20 drops via gastric tube or orally, additionally 2 ml Lefaxine – to be administered by the night-nurse

Care should always be taken of sufficient hydration (orally or via gastric tube)

In patients with elevated serum phosphate: instead of enema, after consultation of the attending physician, 2 Dulcolax and 2 Microlax suppositories should be administered.

# Abdominal (gastrointestinal) surgical patient:

Treatment of constipation only after medical prescription

**3^rd^ postoperative day:** Metoclopramide 10mg IV 3 times daily

**4^th^ postoperative day:** the first enema with foregut after consultation of the attending surgeon
